# Modeling an equivalent b‐value in diffusion‐weighted steady‐state free precession

**DOI:** 10.1002/mrm.28169

**Published:** 2020-01-10

**Authors:** Benjamin C. Tendler, Sean Foxley, Michiel Cottaar, Saad Jbabdi, Karla L. Miller

**Affiliations:** ^1^ Wellcome Centre for Integrative Neuroimaging FMRIB Nuffield Department of Clinical Neurosciences University of Oxford Oxford United Kingdom; ^2^ Department of Radiology University of Chicago Chicago Illinois

**Keywords:** b‐value, diffusion‐weighted spin‐echo, diffusion‐weighted steady‐state free precession, Monte‐Carlo, non‐Gaussian diffusion, postmortem MRI

## Abstract

**Purpose:**

Diffusion‐weighted steady‐state free precession (DW‐SSFP) is shown to provide a means to probe non‐Gaussian diffusion through manipulation of the flip angle. A framework is presented to define an effective b‐value in DW‐SSFP.

**Theory:**

The DW‐SSFP signal is a summation of coherence pathways with different b‐values. The relative contribution of each pathway is dictated by the flip angle. This leads to an apparent diffusion coefficient (ADC) estimate that depends on the flip angle in non‐Gaussian diffusion regimes. By acquiring DW‐SSFP data at multiple flip angles and modeling the variation in ADC for a given form of non‐Gaussianity, the ADC can be estimated at a well‐defined effective b‐value.

**Methods:**

A gamma distribution is used to model non‐Gaussian diffusion, embedded in the Buxton signal model for DW‐SSFP. Monte‐Carlo simulations of non‐Gaussian diffusion in DW‐SSFP and diffusion‐weighted spin‐echo sequences are used to verify the proposed framework. Dependence of ADC on flip angle in DW‐SSFP is verified with experimental measurements in a whole, human postmortem brain.

**Results:**

Monte‐Carlo simulations reveal excellent agreement between ADCs estimated with diffusion‐weighted spin‐echo and the proposed framework. Experimental ADC estimates vary as a function of flip angle over the corpus callosum of the postmortem brain, estimating the mean and standard deviation of the gamma distribution as $1.50\cdot 10^{-4}$ mm^2^/s and $2.10\cdot 10^{-4}$ mm^2^/s.

**Conclusion:**

DW‐SSFP can be used to investigate non‐Gaussian diffusion by varying the flip angle. By fitting a model of non‐Gaussian diffusion, the ADC in DW‐SSFP can be estimated at an effective b‐value, comparable to more conventional diffusion sequences.

## 1 INTRODUCTION

**Figure 1:**
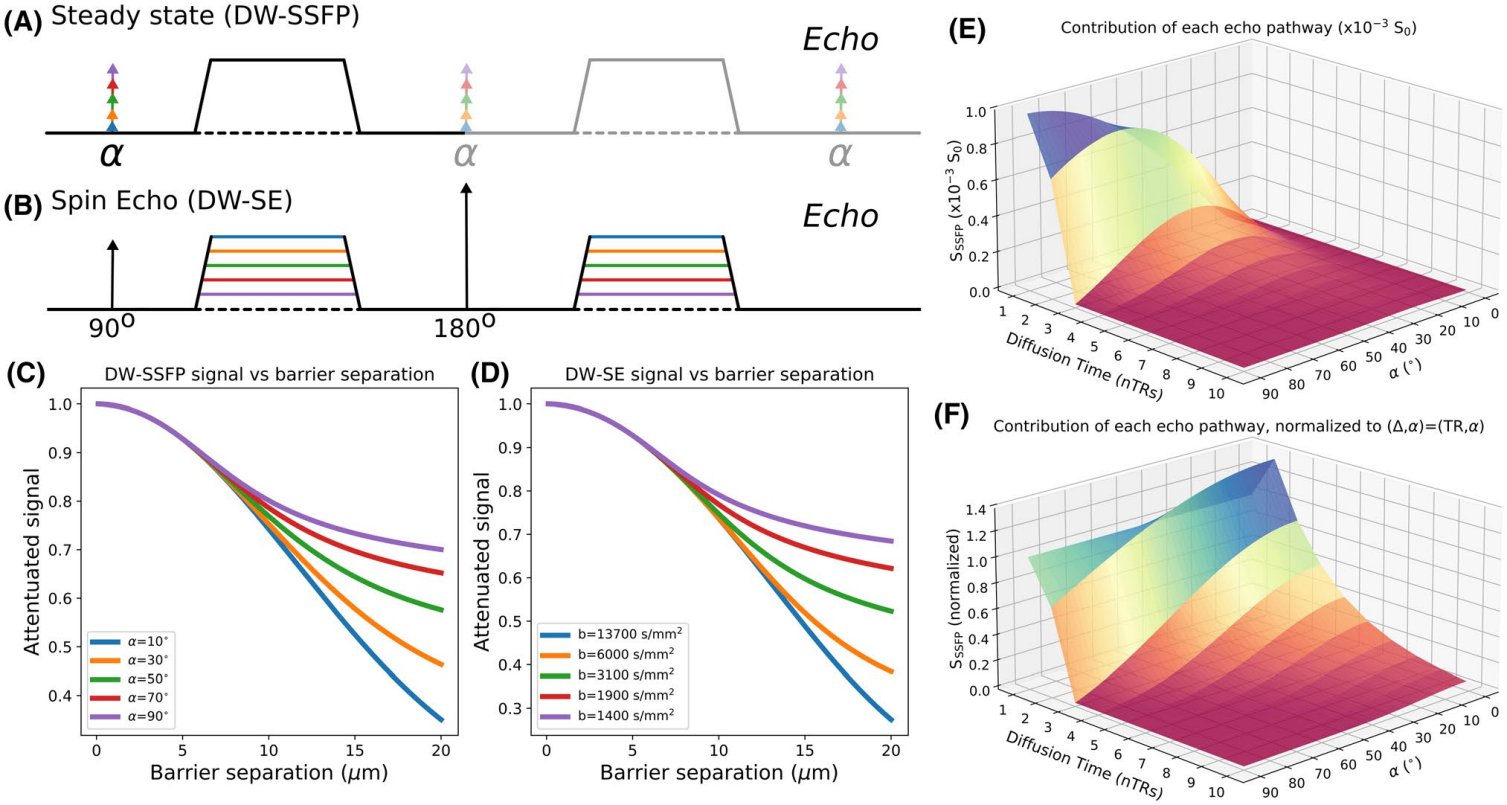
Comparison of DW‐SSFP and DW‐SE sequences under non‐Gaussian diffusion (e.g., due to restricting barriers). A, The DW‐SSFP sequence consists of a single RF pulse and diffusion gradient per TR (this depiction neglects imaging gradients, which are generally refocused to have zero net area) that achieves the equivalent of Stejskal‐Tanner gradient pairs over multiple TR periods. C, In systems with non‐Gaussian diffusion, here represented by diffusion restricted between 2 parallel barriers, changing the flip angle results in a different level of signal attenuation. B, With the DW‐SE sequence, gradient pairs are explicitly included on either side of the refocusing pulse. D, By changing the strength of the applied diffusion gradient, we can achieve a similar sensitivity to restricted diffusion. E, DW‐SSFP signal can be represented as a summation of coherence pathways, each of which has a well‐defined diffusion time representing the number of TR periods between the experienced diffusion gradients. The relative contribution of coherence pathways is a function of flip angle. F, Normalizing these coherence pathways in (E) to the pathway with Δ=TR (i.e., the spin‐echo pathway) provides insight into the sensitivity of DW‐SSFP to non‐Gaussian diffusion. Reducing the flip angle increases the contribution of coherence pathways with longer diffusion times, which are more sensitive to non‐Gaussian diffusion. All simulations performed under the two‐transverse‐period approximation (Equation 1), defining $D=3.5\cdot 10^{-4}$ mm^2^/s, diffusion gradient amplitude = 5.2 G/cm and diffusion gradient duration = 13.56 ms (q = 300 cm^−1^). For the DW‐SE sequence, Δ= 100 ms. For the DW‐SSFP sequence, TR = 28.2 ms, $T_{1}$ = 600 ms and $T_{2}$ = 20 ms. C,D, are simulated with a model of diffusion restricted between 2 parallel barriers as described in Tanner and Stejskal.^16^ E,F, are calculated assuming Gaussian diffusion

Diffusion‐weighted steady‐state free precession (DW‐SSFP) is a powerful sequence that achieves strong diffusion weighting by maintaining a steady‐state in which magnetization accumulates diffusion contrast over multiple repetition times (TRs).^1^, ^2^, ^3^, ^4^ The DW‐SSFP sequence for each TR consists of a single radiofrequency (RF) pulse and single diffusion gradient followed by signal acquisition (Figure 1A). The DW‐SSFP sequence has many favorable properties for probing the diffusion properties of tissue^5^, ^6^: it is very signal‐to‐noise ratio (SNR)‐efficient,^1^, ^7^ generates strong diffusion weighting in MR systems with limited gradient strengths^1^, ^8^, ^9^ and yields high‐SNR diffusivity estimates in samples with short $T_{2}$.^7^, ^10^, ^11^ These properties stem from the steady‐state nature of the sequence.^5^ In DW‐SSFP, transverse magnetization is not spoiled between RF pulses and the short TR (typically TR < $T_{2}$) ensures transverse and longitudinal magnetization persists over multiple excitations, leading to numerous signal‐forming coherence pathways.^12^, ^13^ The signal received from coherence pathways with high b‐values^8^, ^14^ leads to strong diffusion weighting. The short TR prevents relaxation from destroying the available signal before sampling. Although this saturates the magnetization, the large fraction of each TR spent acquiring signal provides a high‐SNR efficiency. Here, we describe the DW‐SSFP variant where the echo is sampled after the diffusion gradient (before the subsequent RF pulse), corresponding to the $M^{-}$ signal in Wu and Buxton.^6^

The DW‐SSFP sequence has 2 major challenges to overcome^5^: first, it is very sensitive to motion; second, it does not have a well‐defined b‐value. One environment where the properties of DW‐SSFP are very well suited is imaging of fixed, postmortem tissue, which is devoid of motion but plagued by low $T_{2}$ and reduced diffusion coefficients.^7^, ^15^ Nevertheless, interpretation of these postmortem data suffer from the lack of a well‐defined b‐value, which is a direct result of the signal reflecting a summation of numerous coherence pathways, each with a different b‐value.^5^, ^8^ As diffusive motion in tissue is generally non‐Gaussian, this poorly‐defined b‐value prevents comparisons between diffusivity estimates obtained with the DW‐SSFP and more conventional measurements using the diffusion‐weighted spin‐echo (DW‐SE) sequence (Figure 1B).

Formation of the steady state in DW‐SSFP is a function of both experimental parameters (flip angle and TR) and sample properties (relaxation and diffusivity). However, unlike the DW‐SE sequence, the diffusion‐weighted terms in DW‐SSFP are not readily separable as a simple multiplicative term.^8^ Instead, alterations in the prescribed flip angle, TR, and relaxation times alter the relative weighting of each coherence pathway and, hence, result in a different diffusion weighting.^5^ This surprising result highlights the fact that in DW‐SSFP, there is not a standalone diffusion preparation (gradients and their timings) that determines the degree of diffusion weighting, as is the case for spin‐ and stimulated‐echo sequences.

Looking at this from a different perspective, the idiosyncrasies of the DW‐SSFP signal formation mechanism present us with an opportunity: to probe the diffusion properties of tissue without any modification to the diffusion encoding gradients. Figure 1C, D simulates the received DW‐SSFP and DW‐SE signal for diffusion restricted between 2 parallel barriers.^16^ Signal attenuation is altered by changing the flip angle in DW‐SSFP (Figure 1C), similar to changing the b‐value in DW‐SE (Figure 1D).

In this work, we show that we can probe different diffusion time (and, therefore, b‐value) regimes by varying the flip‐angle in DW‐SSFP. As with varying b‐values in more conventional diffusion measurements, this flip angle dependence changes the apparent diffusion coefficient (ADC) estimates in systems with non‐Gaussian diffusion. Based on this concept, we propose a method to translate quantitative diffusivity estimates derived with DW‐SSFP, in which b‐values are not well defined, into ADC estimates at a single effective b‐value, as would be measured using more conventional sequences such as DW‐SE. This is achieved by defining DW‐SSFP signal behavior under a model of non‐Gaussianity and translating the measured DW‐SSFP signal at multiple flip angles into an ADC at an equivalent, well‐defined b‐value. The specific model presented here combines a gamma variate distribution of diffusivities with the Buxton model of DW‐SSFP signal,^8^ but can be adapted to other forms of non‐Gaussianity^17^ and alternative signal models.^4^, ^18^ The derived signal model is verified with Monte‐Carlo simulations of both DW‐SSFP and DW‐SE signal evolution, and the expected signal dependence is demonstrated using DW‐SSFP datasets acquired at multiple flip angles in postmortem brain tissue.

## 2 THEORY

### 2.1 Two‐transverse‐period approximation

The two‐transverse‐period approximation of DW‐SSFP^6^, ^8^ is a signal model that makes the simplifying assumption that coherence pathways do not survive beyond 2 periods in the transverse plane. This approximation, considered valid when TR ≥ ~1.5 ⋅ $T_{2}$,^8^ is particularly helpful for building intuition into the dependence of diffusion times on flip angle. Under these conditions, the DW‐SSFP signal can be described as the weighted sum of spin‐ and stimulated‐echo pathways:

(1) $$S_{\text{SSFP}}\left(\alpha ,\;T_{1},\;T_{2},\;\text{TR},\;q,\;D\right)\;=\;\frac{-S_{0}\left(1-E_{1}\right)E_{1}E_{2}^{2}\sin\alpha }{2\left(1-E_{1}\cos\alpha \right)}\cdot \left[\underset{\text{Spin echo}}{\underbrace{\frac{1-\cos\alpha }{E_{1}}A_{1}}}\;+\;\sin^{2}\alpha \underset{\text{Stimulated echoes}}{\underbrace{\sum_{n=1}^{\infty }{\left(E_{1}\cos\alpha \right)}^{n-1}A_{1}^{n+1}}}\right],$$

where $S_{0}$ is the equilibrium magnetization, $E_{1}=e^{-\frac{\text{TR}}{T_{1}}}$, $E_{2}=e^{-\frac{\text{TR}}{T_{2}}}$ , α is the flip angle, $A_{1}=e^{-q^{2}\cdot \text{TR}\cdot D}$ (D is the diffusion coefficient) and q=γGτ (γ is the gyromagnetic ratio, G is the diffusion gradient amplitude and τ is the diffusion gradient duration). In Equation 1, the first term in the square brackets represents a spin‐echo pathway (i.e., the magnetization that is in the transverse plane in 2 consecutive TRs), and the second term describes the stimulated‐echo pathways (characterized by 2 transverse periods separated by *n* longitudinal periods). The diffusion time, Δ, is well defined for each individual pathway (spin echo: Δ=TR, stimulated echo: $\Delta =\left(n+1\right)\cdot \text{TR}$). The effect of diffusion time is embodied in A_1_, with each pathway attenuated by $e^{-q^{2}\cdot \Delta \cdot D}$. Under the two‐transverse‐period approximation, the signal is a weighted sum of contributions from different pathways with different diffusion times, with relative signal weights that depend on the flip angle (α), TR and $T_{1}$. Changes in $T_{2}$ do not alter the relative weighting of each pathway, because the assumption is that only coherence pathways with 2 transverse periods contribute to the signal. Example pathways are illustrated in Supporting Information Figure S1A‐C, which is available online.

Figure 1E visualizes the signal contributions of each pathway (amplitudes calculated from individual terms in the summation in Equation 1). Pathways with longer diffusion times lead to signals that are more diffusion weighted and informative about restrictive diffusion. At intermediate flip angles the overall signal contribution from the different pathways peaks, leading to increased SNR. We can visualize the relative contributions of different pathways at a given flip angle by normalizing to the signal from the spin‐echo (Δ=1·TR) pathway (Figure 1F). This normalization makes it clear that decreasing the flip angle increases the relative contribution of simulated‐echo pathways with longer diffusion times, leading to an increase in diffusion contrast. However, this comes at a tradeoff with overall signal levels (Figure 1E).^7^

The two‐transverse‐period approximation provides an intuitive way to see that changing the flip angle in DW‐SSFP alters the diffusion time regime that the signal is sensitive to, with an increased flip angle corresponding to a shorter effective diffusion time. The DW‐SSFP signal can be thought as a temporally blurred mixture of the “cleaner” diffusion time behavior that is captured by more conventional DW‐SE (or diffusion‐weighted stimulated‐echo) signals, corresponding to a single point on the Δ axis.

### 2.2 Full Buxton model of DW‐SSFP

The full Buxton model of DW‐SSFP^6^, ^8^ accounts for all coherence pathways, including those that survive more than 2 TRs in the transverse plane. Summing over all coherence pathways yields the expression:

(2) $$S_{\text{SSFP}}\left(\alpha ,\;T_{1},\;T_{2},\;\text{TR},\;q,\;D\right)=-\frac{S_{0}\left(1-E_{1}\right)E_{2}A_{2}^{-\frac{2}{3}}\left(F_{1}-E_{2}A_{1}A_{2}^{\frac{2}{3}}\right)\sin\alpha }{r-F_{1}s},$$

where $A_{2}=e^{-q^{2}\cdot \tau \cdot D}$. Definitions of r, s, and $F_{1}$ are provided in the Appendix. This more complete model allows for the existence of additional coherence pathways, including pathways that remain in the transverse plane over multiple TRs, and coherence pathways that give rise to multiple signal forming echoes over their lifetime.^14^ This leads to pathways experiencing more than 2 diffusion gradients, including some with a q‐value that is an even multiple of the q in a single TR period. Here, the relative signal weighting of pathways is additionally dependent on $T_{2}$.^14^ Examples of these additional pathways are given in Supporting Information Figure S1. Under the full Buxton model, we, therefore, lose a strict correspondence between pathway and diffusion time; instead, changing the flip angle is equivalent to probing different b‐value regimes, with smaller effective b‐value at higher flip angle. The DW‐SSFP signal is a blurred mixture of the “cleaner” b‐value behavior that is captured by more conventional DW‐SE (or diffusion‐weighted stimulated‐echo) signals. As with spin‐echo measurements, calculating an ADC with DW‐SSFP requires estimates of both diffusion‐weighted and non–diffusion‐weighted signals (with the caveat in DW‐SSFP that a small gradient is still required to avoid banding patterns associated with fully‐balanced SSFP),^19^ in addition to estimates of $T_{1}$ and $T_{2}$.

### 2.3 Investigating Non‐Gaussianity

Diffusion in tissue is restricted and hindered by membranes, causing the ADC at higher b‐values to be less than one would predict using the Gaussian propagator describing free diffusion. As can be inferred from Figure 1C‐F, non‐Gaussianities, which are typically observed as a dependence of diffusivity on b‐value, will give rise to variable apparent diffusion coefficients (ADCs) for different flip angles in DW‐SSFP. Hence, while conventional sequences typically characterize non‐Gaussian diffusion using measurements at multiple diffusion times or q‐values, this can also be accomplished in DW‐SSFP through measurements at multiple flip angles. This also provides a route to address the poorly defined b‐value in a DW‐SSFP measurement, through translation into a more conventional framework with a well‐defined b‐value.

**Figure 2:**
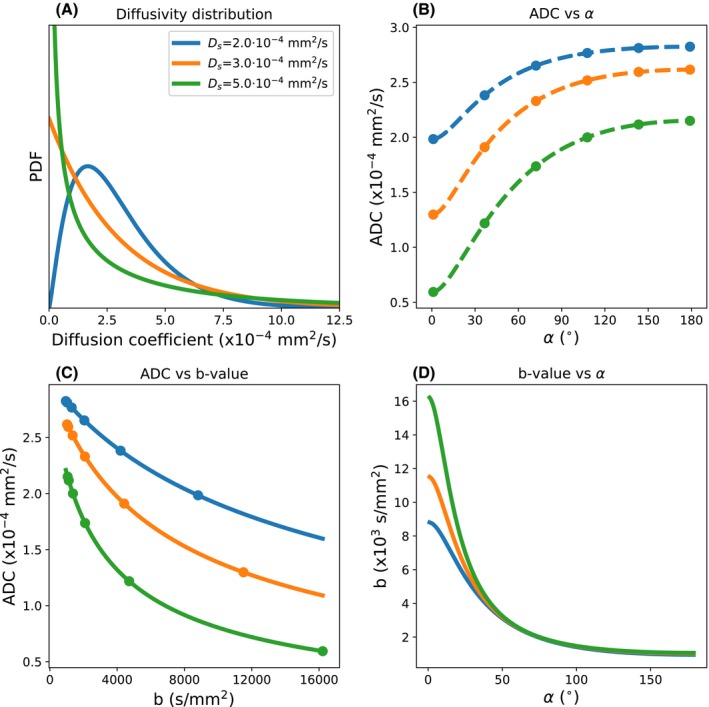
A, Three different gamma distributions with $D_{m}=3.0\cdot 10^{-4}$ mm^2^/s and $D_{s}$ defined as per the legend. B, The associated evolution of ADC with flip angle for DW‐SSFP under these distributions. The Buxton model for Gaussian diffusion can be fit to DW‐SSFP measurements (B, dots) to obtain ADC estimates at multiple flip angles. Comparing Equations 2 and 4, we can then fit a gamma distribution to these ADC estimates (B, dashed lines). C, If we wish to translate this gamma variate to the equivalent ADC estimates that would be obtained from DW‐SE, we can subsequently calculate the ADC for a given b‐value assuming the same gamma distribution (solid lines). D, Alternatively, we can define a DW‐SE b‐value at any given DW‐SSFP flip angle that gives rise to an equivalent ADC. Combining these expressions, we can plot the ADC estimates measured with DW‐SSFP (B, dots) versus the DW‐SE b‐value (C, dots). Simulation performed over the range α= 1°‐179°, setting the diffusion gradient amplitude = 5.2 G/cm & diffusion gradient duration = 13.56 ms (q = 300 cm^−1^), TR = 28.2 ms, $T_{1}$ = 600 ms, and $T_{2}$ = 20 ms. To eliminate the effects of $S_{0}$, the ADC was estimated at each flip angle (B) by fitting to the diffusion‐weighted signal divided by the non–diffusion‐weighted signal

We demonstrate this concept using a gamma distribution of diffusivities (Figure 2A) to describe non‐Gaussian diffusion.^20^, ^21^ The gamma distribution, $\rho \left(D;\;D_{m},\;D_{s}\right)$, can be described in terms of a mean, $D_{m}$, and a standard deviation, $D_{s}$. For DW‐SE, the signal for a gamma distribution of diffusivities is defined as^20^, ^21^:

(3) $$\begin{array}{c} S_{\text{SE},\Gamma }\left(b,\;D_{m},\;D_{s}\right)=S_{0}\int_{0}^{\infty }e^{-bD}\rho \left(D;\;D_{m},\;D_{s}\right)dD\\ \;=S_{0}{\left(\frac{D_{m}}{D_{m}+bD_{s}^{2}}\right)}^{\frac{D_{m}^{2}}{D_{s}^{2}}}, \end{array}$$

where $b=q^{2}\cdot \left(\Delta -\tau /3\right)$. This distribution of diffusivities can be embedded in the full Buxton signal model of DW‐SSFP as:

(4) $$\begin{array}{c} S_{\text{SSFP},\Gamma }\left(\alpha ,\;T_{1},\;T_{2},\;\text{TR},\;q,\;D_{m},\;D_{s}\right)=\\ -S_{0}\left(1-E_{1}\right)E_{2}\sin\alpha \int_{0}^{\infty }\frac{A_{2}^{-2/3}\left(F_{1}-E_{2}A_{1}A_{2}^{2/3}\right)}{r-F_{1}s}\rho \left(D;\;D_{m},\;D_{s}\right)dD. \end{array}$$

This integral can be evaluated using numerical integration. Figure 2B depicts how the ADC varies (fitting process described in the following section) as a function of flip angle for 3 different gamma distributions (Figure 2A). As we increase the flip angle, we obtain a higher estimate of ADC, consistent with our expectations of an increased ADC estimate as we decrease the b‐value.

### 2.4 A framework to translate between DW‐SSFP and DW‐SE measurements

Given quantification of the ADC in DW‐SSFP, we can define an ‘effective’ b‐value to be that which yields the same ADC estimate using the DW‐SE sequence. Translating ADC estimates from DW‐SSFP into an equivalent ADC at a single b‐value can be achieved in the context of a common, underlying non‐Gaussianity.

From DW‐SSFP data obtained at multiple flip angles, the ADC can be uniquely determined at each flip angle by solving Equation 2 (Figure 2B, dots), given knowledge of the experimental protocol, $T_{1}$, $T_{2}$, and non–diffusion‐weighted DW‐SSFP data (to estimate $S_{0}$). Our diffusion model (Figure 2A) can be subsequently fitted to the multi‐flip data (Equations 2 and 4) to uniquely determine a value of $D_{m}$ and $D_{s}$ that can describe the evolution of ADC with flip angle (Figure 2B, dashed lines). We can use the values of $D_{m}$ and $D_{s}$ to subsequently simulate the ADC at any given DW‐SE b‐value (Figure 2C) by comparing Equation 3 with the DW‐SE signal under the Stejskal‐Tanner model ($S=S_{0}\exp\left(-bD\right)$). Alternatively, we can determine the equivalent b‐value that would yield the same estimate of ADC as measured with DW‐SSFP (Figure 2D) at a given flip angle. A detailed processing pipeline is provided in Supporting Information Figure S2.

## 3 METHODS

### 3.1 Monte‐Carlo simulations of DW‐SSFP and DW‐SE signal

Uniformly distributed spin trajectories were generated using Camino^22^ ($5\cdot 10^{5}$ spins, $D=3.5\cdot 10^{-4}$ mm^2^/s, 250 time steps), modified to produce trajectories that followed a Gaussian distribution of displacements per time‐step.^22^ A gamma distribution of diffusivities was subsequently generated from the trajectories using MATLAB (version 2017a, The MathWorks, Inc., Natick, MA) by scaling the displacement of individual spin trajectories to modify their diffusion coefficient (D) to correspond to a gamma distribution when considering the spin ensemble. Here, we set $D_{m}=1.50\cdot 10^{-4}$ mm^2^/s and $D_{s}=2.10\cdot 10^{-4}$ mm^2^/s for our simulated gamma distribution, consistent with the corpus callosum of the postmortem brain used in our experiment (see following section and the Results section).

The DW‐SSFP signal was simulated using in‐house code written in MATLAB, with approximately the same parameters as used in our experimental measurements (TR = 28.2 ms, τ = 13.56 ms, G = 52 mT/m, q = 300 cm^−1^, flip angles = 10° to 170° in 10°‐increments), setting $T_{1}=$ 568 ms and $T_{2}=$ 19.8 ms, the mean over the corpus callosum of the postmortem brain. Non–diffusion‐weighted DW‐SSFP data were additionally simulated, setting D=0 mm^2^/s. A single time step corresponded to 1 TR.

A DW‐SE signal was additionally simulated (τ = 13.56 ms, Δ=40 ms, b‐values = 0 to 14,000 s/mm^2^ at 1000 s/mm^2^ increments, achieved by changing G). A single time step corresponds to 0.4 ms.

### 3.2 Experimental demonstration of DW‐SSFP flip angle dependency

A whole postmortem brain was scanned on a 7T Siemens MR system (1Tx/32Rx head coil) with a DW‐SSFP sequence for a single diffusion direction at multiple flip angles (resolution = 0.85 × 0.85 × 0.85 mm^3^, TR = 28.2 ms, echo time = 21 ms, bandwidth = 393 Hz/pixel, τ = 13.56 ms, G = 52 mT/m, q= 300 cm^−1^, direction = [0.577, 0.577, 0.577], flip angles = 10° to 90° at 5°‐increments). At each flip angle, an equivalent non–diffusion‐weighted DW‐SSFP dataset was acquired with a small diffusion gradient (q= 20 cm^−1^) to ensure dephasing of the magnetization and to prevent banding artefacts.^19^ $T_{1}$, $T_{2}$, and $B_{1}$ maps^23^ were additionally acquired (details of acquisition and processing provided in Supporting Information Table S1) over the postmortem brain, which are required for accurate modeling of the DW‐SSFP signal.^11^

A Gibbs ringing correction was applied to the DW‐SSFP images.^24^ To reduce noise floor bias, the mean background signal was estimated and removed from the DW‐SSFP signal.^25^ All coregistrations were performed using a 6 degrees‐of‐freedom transformation with FLIRT.^26^, ^27^

The voxelwise ADC was estimated over the corpus callosum at each flip angle using Equation 2. To eliminate the effects of $S_{0}$, the experimental diffusion‐weighted DW‐SSFP data were normalized by the non–diffusion‐weighted DW‐SSFP data and fit with $S_{\text{SSFP}}\left(\alpha ,\;T_{1},\;T_{2},\;\text{TR},\;q,\;\text{ADC}\right)/S_{\text{SSFP}}\left(\alpha ,\;T_{1},\;T_{2},\;\text{TR},\;0,\;\text{ADC}\right)$ (noting $S_{\text{SSFP}}\left(\alpha ,\;T_{1},\;T_{2},\;\text{TR},\;0,\;\text{ADC}\right)\neq S_{0})$. The mean ADC over the corpus callosum was subsequently calculated at each flip angle and fit to Equation 4 to determine $D_{m}$ and $D_{s}$. Fitting was performed in Python^28^ using the SciPy *curve_fit* function, implemented with the Levenberg‐Marquardt algorithm.^29^ Numerical integration of Equation 4 was performed using the SciPy *quad* command.

## 4 RESULTS

### 4.1 Monte‐Carlo simulations of the DW‐SSFP and DW‐SE sequence

**Figure 3:**
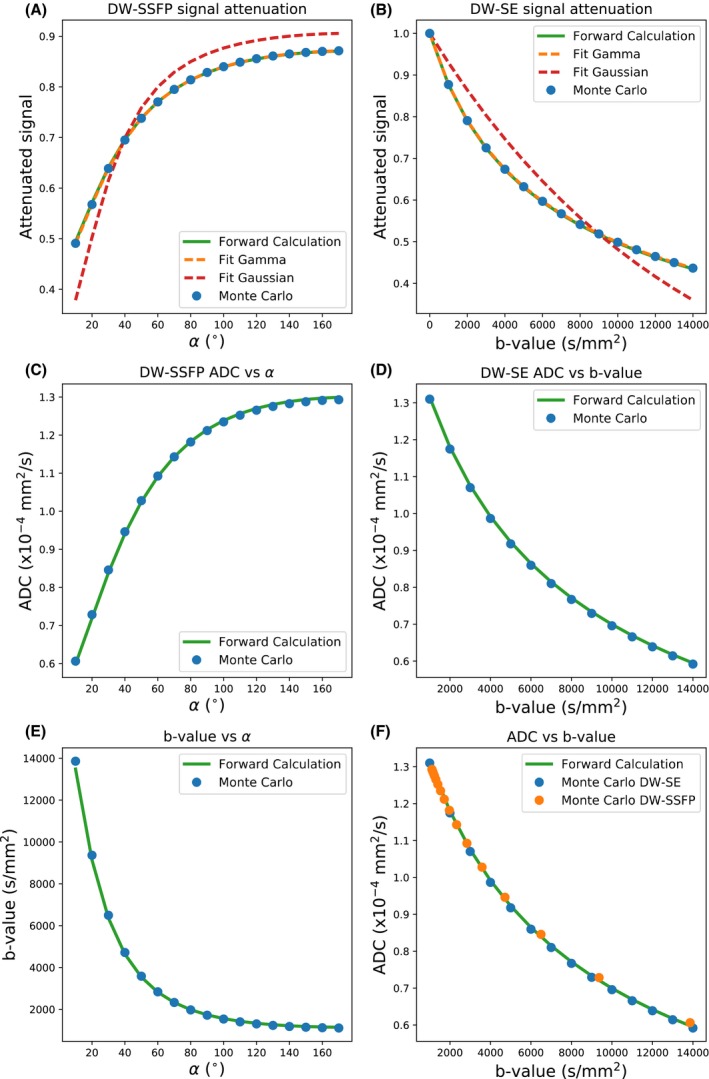
Results of the Monte‐Carlo simulations. A,B, reveal the signal attenuation for the DW‐SSFP (A) and DW‐SE (B) simulations, respectively: Monte‐Carlo simulations (blue dots), forward calculations of the DW‐SSFP and DW‐SE signal under a gamma‐variate distribution (green lines), fits to the Monte‐Carlo solutions (dashed orange lines), and fits assuming only a single diffusion coefficient (dashed red lines). C, D, reveal how the estimated ADC varies with DW‐SSFP flip angle and DW‐SE b‐value. By comparing the ADC estimates in (C) and (D), we can determine which DW‐SSFP flip angle gives rise to an equivalent ADC estimate (E). F, This allows us to transform our Monte‐Carlo estimates of ADC with the DW‐SSFP sequence into the same space as the DW‐SE sequence

Figure 3A,B compares the simulated signal attenuation of the DW‐SSFP and DW‐SE signal estimated for a gamma‐variate distribution (blue circles) to forward calculations from Equations 3 and 4 (green lines). Fitting to the Monte‐Carlo signals, we estimated $D_{m}=1.48\cdot 10^{-4}$ mm^2^/s and $D_{s}=2.04\cdot 10^{-4}$ mm^2^/s for DW‐SSFP, and $D_{m}=1.49\cdot 10^{-4}$ mm^2^/s and $D_{s}=2.10\cdot 10^{-4}$ mm^2^/s for DW‐SE (original values $D_{m}=1.50\cdot 10^{-4}$ mm^2^/s and $D_{s}=2.10\cdot 10^{-4}$ mm^2^/s). Similar to acquiring DW‐SE data at multiple b‐values, these simulations suggest that the DW‐SSFP signal acquired at multiple flip angles is able to encode non‐Gaussian diffusion. Fitting a Gaussian model assuming a single diffusion coefficient (red line) is unable to provide an accurate fit.

By calculating ADC estimates from the signal attenuation using the full Buxton model for DW‐SSFP and the Stejskal‐Tanner model for DW‐SE (i.e., both assuming purely Gaussian diffusion, shown in Figure 3C,D), we can determine the equivalent DW‐SE b‐value that corresponds to the ADC estimate at each DW‐SSFP flip angle (Figure 3E). These results highlight the substantial range of effective b‐values achievable with the DW‐SSFP sequence by modifying the flip angle alone. With this, we are able to translate our DW‐SSFP signal, which reflects a blurring of different signals with well‐defined b‐values, into a DW‐SSFP ADC at a well‐defined effective b‐value, demonstrating the same ADC evolution as DW‐SE data (Figure 3F). Note that in Figure 3F, orange and blue data points were derived from separate simulations.

### 4.2 Experimental validation of DW‐SSFP flip angle dependency

**Figure 4:**
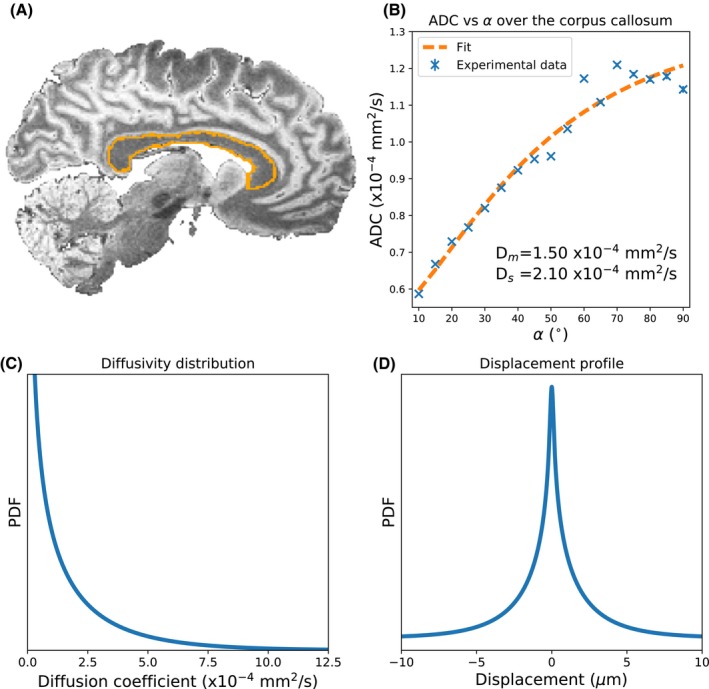
A, Sagittal slice of the DW‐SSFP data (q = 20 cm^−1^) acquired over the postmortem brain with the corpus callosum outlined in orange. B, By determining the ADC at each flip angle (blue crosses) using Equation 2, we demonstrate that the flip angle in DW‐SSFP sensitizes us to different b‐value regimes, leading to changing ADC estimates. Fitting our gamma distribution model (Equation 4) to the experimental data (B, orange dashed line), we estimate $D_{m}=1.50\cdot 10^{-4}$ mm^2^/s and $D_{s}=2.10\cdot 10^{-4}$ mm^2^/s in our postmortem brain, with (C) and (D) displaying the resulting diffusivity distribution and displacement profile for these parameters. B, Error bars display the standard error of the ADC over the corpus callosum, but are not visible for most flip angles

Figure 4 reveals the variation in ADC over the corpus callosum (Figure 4A) of the postmortem brain (blue crosses in Figure 4B), where the ADC estimated at 90° is almost twice the ADC estimate at 10°, despite no changes in the diffusion encoding of the sequence. This variation is consistent with non‐Gaussian diffusion, and inconsistent with Gaussian diffusion, which would correspond to a flat line in Figure 4B. By fitting the ADC estimates to a gamma distribution (dashed orange line), we estimated $D_{m}=1.50\cdot 10^{-4}$ mm^2^/s and $D_{s}=2.10\cdot 10^{-4}$ mm^2^/s. The corresponding probability density function and displacement profile are shown in Figure 4C, D.

## 5 DISCUSSION

The DW‐SSFP signal represents a blurred mixture of signals with well‐defined b‐values. By defining DW‐SSFP derived ADC estimates in terms of an effective b‐value, we can transform these estimates into alignment with more conventional diffusion measurements. In the context of postmortem imaging, this could for example facilitate comparisons of diffusivity estimates acquired in postmortem tissue with DW‐SSFP to in vivo diffusivity estimates acquired with DW‐SE. Monte‐Carlo simulations (Figure 3) yield excellent agreement between simulated signals for a given gamma‐distributed system and our forward model. These results suggest that the DW‐SSFP signal is able to capture non‐Gaussianity and verify the ability to transform DW‐SSFP signals into equivalent DW‐SE signals. Experimental fitting of our model to data acquired in in the corpus callosum of a whole postmortem brain (Figure 4) demonstrates that use of the original Buxton model produces the predicted flip‐angle dependence of the ADC estimate that is expected for non‐Gaussian diffusion. Our gamma‐distribution model fit (Figure 4B – dashed orange line) is able to explain this flip‐angle dependence of ADC.

The observation of flip‐angle‐based sensitivity to non‐Gaussian diffusion also suggests challenges to the use of DW‐SSFP. Previous work has demonstrated that the DW‐SSFP sequence yields improved SNR at 7T versus 3T in postmortem tissue with short $T_{2}$, motivating its use at ultra‐high field.^11^ However, $B_{1}$‐inhomogeneity (e.g., at ultra‐high field) translates into varying effective b‐value across a sample, leading to spatially varying ADC estimates even when the underlying tissue properties are the same. This confound prevents a simple interpretation of results between, or even within DW‐SSFP datasets. One approach is to use the model parameters to derive an ADC map with the same effective b‐value within every voxel regardless of local $B_{1}$, representing a common snapshot of restricted diffusion.^30^ This approach could additionally account for the variations in $T_{1}$, $T_{2}$, and the diffusivity of tissue, which will also influence the effective b‐value (see Supporting Information Figure S2).

**Figure 5:**
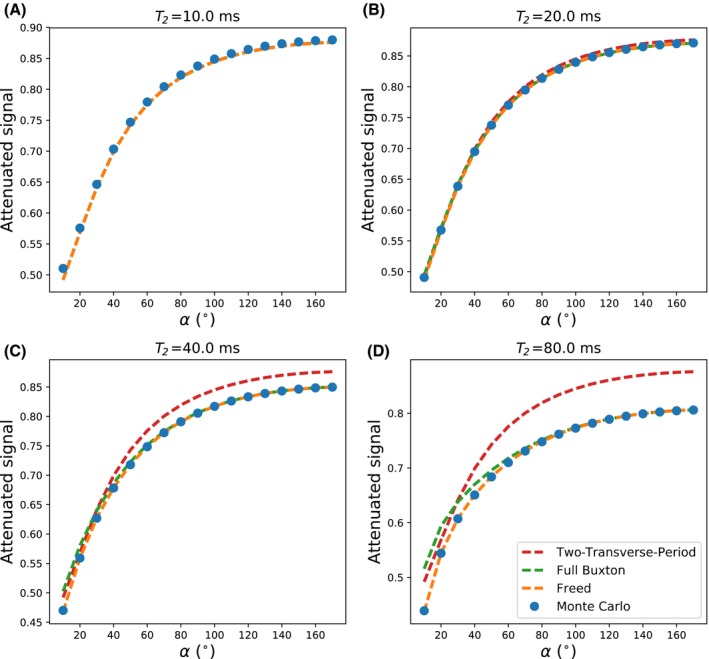
A‐D, Attenuation of the DW‐SSFP signal for 4 different values of $T_{2}$, comparing the signal attenuation from Monte‐Carlo simulations (blue dots) versus analytical solutions of the two‐transverse‐period (red dashed line),^8^ full Buxton (green dashed line),^8^ and the Freed (orange dashed line)^18^ model assuming a gamma distribution of diffusivities. Simulated parameters (except for $T_{2}$) are identical to the Monte‐Carlo simulations described in the main text. As the $T_{2}$ estimate increases, we observe a substantial deviation of the signal attenuation predicted by the two‐transverse‐period model versus the Monte‐Carlo estimates. Similarly, a deviation is seen with the Full Buxton model, particularly at lower flip angles. As described by Freed et al,^18^ under certain experimental regimes the full Buxton model no longer provides accurate estimates of the DW‐SSFP signal. The Freed model, however, provides excellent agreement to the Monte‐Carlo simulations across the range of $T_{2}$ values simulated

An early version of this framework used the two‐transverse‐period approximation of DW‐SSFP (Equation 1) to derive analytical solutions (see Appendix) for the ADC and signal under a gamma distribution.^31^ However, further analysis with Monte‐Carlo simulations revealed substantial deviations in signal attenuation when the two‐transverse‐period condition (TR ≥ ~1.5 ⋅ $T_{2}$) is violated (Figure 5). By using numerical integration, we can incorporate other diffusivity distributions without analytical solutions. Moreover, at longer $T_{2}$, the gamma‐distributed Buxton model deviates from Monte‐Carlo simulations, whereas the Freed model^18^ provided excellent agreement (Figure 5). In general, the framework presented here is compatible with any DW‐SSFP signal model and could be extended to other models of non‐Gaussianity. Furthermore, this approach could be extended to other DW‐SSFP variants, e.g., the diffusion‐weighted signal before the diffusion gradient (corresponding to the $M^{+}$ signal in Wu and Buxton^6^), or even more complicated combinations of echoes.^32^

Subsequent to designing this protocol, we appreciated that the use of q = 20 cm^−1^ in our non–diffusion‐weighted DW‐SSFP datasets provides less than 2π phase across a voxel, meaning that the higher‐order pathways are not completely dephased and thus will contribute some net signal. However, the fact that positive and negative pathways of the same order have opposing phase will lead to partial cancellation. This will have only affected the low q‐value data in our case, and would result in a spatially varying bias in $S_{0}$, which we eliminate through normalization before fitting. In addition, the Monte‐Carlo simulations were performed with 2π phase across the simulated voxel, with results consistent with those obtained within our postmortem experiment and our proposed framework.

One limitation of our study is the lack of comparison between experimental DW‐SSFP and DW‐SE data. Such a comparison would require acquisition of both DW‐SSFP and DW‐SE data at multiple flip angles/b‐values. However, DW‐SE measurements in postmortem tissue suffer from very low SNR and are beyond the scope of this study.

## 6 CONCLUSIONS

By acquiring DW‐SSFP data at multiple flip angles, we can probe the non‐Gaussian diffusion properties of a sample. We can additionally disentangle the blurred mixture of diffusion‐weighted signals with different b‐values in DW‐SSFP. This approach enables the transformation of ADC estimates derived from DW‐SSFP to more conventional sequences at a single effective b‐value.

## Supporting information


**FIGURE S1** In DW‐SSFP, repeated application of RF pulses decomposes the magnetization into a series of coherence pathways, which are sensitized to the diffusion gradient during transverse periods. Here we show 5 example coherence pathways. The spin‐echo pathway (A), stimulated‐echo pathway (B), and long stimulated‐echo pathway (C) only survive for 2 TRs in the transverse plane, the condition for the two‐transverse‐period approximation (1). These pathways all experience the same q‐value, but have different diffusion times, defined as Δ=1·TR (A), 2·TR (B), and 4·TR (C). For the full Buxton model (1), this condition is no longer required, and pathways can experience cumulative sensitization to the diffusion gradients over multiple TRs, such as the spin‐echo pathway in (D), in addition to pathways that generate multiple echoes over their lifetime (E). This leads to pathways with different q‐values, in addition to weighting of the signal by $T_{2}$
**FIGURE S2** Processing pipeline for (A) 2 samples with different diffusion properties but identical relaxation times and (B) identical diffusion properties but different $T_{1}$ values. Experimental DW‐SSFP data are acquired at multiple flip angles (i, dots) and converted into ADC estimates (ii, dots) (Equation 2, main text). To eliminate the effects of $S_{0}$, we fit to the DW‐SSFP signal attenuation. The DW‐SSFP signal model incorporating a gamma distribution of diffusivities (Equation 4, main text) is subsequently fit to the ADC estimates at multiple flip angles (by comparing to Equation 2, main text) to determine $D_{m}$ and $D_{s}$ (iii). From Equation 3 in the main text and our fitted values of $D_{m}$ and $D_{s}$, we can simulate the estimated ADC with b‐value for a DW‐SE sequence by making comparisons with the DW‐SE signal under the Stejskal‐Tanner model ($S=S_{0}\exp\left(-bD\right)$). From this, we can define an equivalent DW‐SE b‐value, which gives rise to the same ADC estimate at each DW‐SSFP flip angle (iv). Our ADC estimates with DW‐SSFP can be subsequently plotted versus an effective b‐value (v). In (A), this leads to distinct evolution of ADC with effective b‐value for the 2 samples (v). However, in (B), the signal evolution is identical (v), despite having a different ADC evolution versus flip angle (ii), reflecting differences in the weighting of the different coherence pathways due to relaxation, leading to different effective b‐values along the ADC curve (v, dots)
**TABLE S1** Acquisition protocols for the $T_{1}$, $T_{2}$ and $B_{1}$ maps. Before processing, a Gibbs ringing correction was applied to the TIR and TSE data (2). $T_{1}$ and $T_{2}$ maps were derived assuming mono‐exponential signal evolution. The $B_{1}$ map was obtained using the methodology described in (3)

## Appendix 1

### Full Buxton model definitions

(A1) $$\begin{array}{c} r=1-E_{1}\cos\alpha +E_{2}^{2}A_{1}A_{2}^{1/3}\left(\cos\alpha -E_{1}\right),\\ s=E_{2}A_{1}A_{2}^{-4/3}\left(1-E_{1}\cos\alpha \right)+E_{2}A_{2}^{-1/3}\left(\cos\alpha -E_{1}\right),\\ F_{1}=K-{\left(K^{2}-A_{2}^{2}\right)}^{1/2},\\ K=\frac{1-E_{1}A_{1}\cos\alpha -E_{2}^{2}A_{1}^{2}A_{2}^{-2/3}\left(E_{1}A_{1}-\cos\alpha \right)}{E_{2}A_{1}A_{2}^{-4/3}\left(1+\cos\alpha \right)\left(1-E_{1}A_{1}\right)}. \end{array}$$

### Two‐transverse‐period approximation of ADC and signal under a gamma distribution

Under the two‐transverse‐period approximation of DW‐SSFP (Equation 1), we can define:

(A2) $$\begin{array}{c} {\text{ADC}}_{\text{SSFP}}=\\ \;-\frac{1}{q^{2}\text{TR}}\cdot \ln\left[\frac{-\left(S_{\text{SSFP}}^{{}^{\prime }}\cdot E_{1}\cos\alpha +1\right)+{\left[{\left(S_{\text{SSFP}}^{{}^{\prime }}\cdot E_{1}\cos\alpha +1\right)}^{2}+4E_{1}\cdot S_{\text{SSFP}}^{{}^{\prime }}\right]}^{\frac{1}{2}}}{2E_{1}}\right], \end{array}$$

where:

(A3) $$S_{\text{SSFP}}^{{}^{\prime }}=\frac{S_{\text{SSFP}}\left(\alpha ,T_{1},T_{2},\text{TR},q,\text{ADC}\right)}{S_{\text{SSFP}}\left(\alpha ,T_{1},T_{2},\text{TR},0,\text{ADC}\right)}\cdot \frac{1+E_{1}}{1-E_{1}\cos\alpha }.$$

For a gamma distribution:

(A4) $$\begin{array}{c} S_{\text{SSFP},\Gamma }^{{}^{\prime }}={\left(\frac{D_{m}}{D_{m}+q^{2}\cdot \text{TR}\cdot D_{s}^{2}}\right)}^{\frac{D_{m}^{2}}{D_{s}^{2}}}+\\ E_{1}\cdot \left(1+\cos\alpha \right)\cdot {\left(\frac{D_{m}}{q^{2}\cdot \text{TR}\cdot D_{s}^{2}}\right)}^{\frac{D_{m}^{2}}{D_{s}^{2}}}\cdot \Phi \left(E_{1}\cos\alpha ,\frac{D_{m}^{2}}{D_{s}^{2}},\frac{D_{m}}{q^{2}\cdot \text{TR}\cdot D_{s}^{2}}+2\right), \end{array}$$

where Φ is the Lerch transcendent.^33^ Derivations are provided in Supporting Information.
