# Supplementary material for: Modeling an equivalent b‐value in diffusion‐weighted steady‐state free precession
Source: Magn Reson Med. 2020 Jan 10;84(2):873–84. doi: 10.1002/mrm.28169 (PMC7216928; doi:10.1002/mrm.28169)
Supplement: Supplementary file 1 — FIGURE S1 In DW‐SSFP, repeated application of RF pulses decomposes the magnetization into a series of coherence pathways, which are sensitized to the diffusion gradient during transverse periods. Here we show 5 example coherence pathways. The spin‐echo pathway (A), stimulated‐echo pathway (B), and long stimulated‐echo pathway (C) only survive for 2 TRs in the transverse plane, the condition for the two‐transverse‐period approximation (1). These pathways all experience the same q‐value, but have different diffusion times, defined as Δ=1·TR (A), 2·TR (B), and 4·TR (C). For the full Buxton model (1), this condition is no longer required, and pathways can experience cumulative sensitization to the diffusion gradients over multiple TRs, such as the spin‐echo pathway in (D), in addition to pathways that generate multiple echoes over their lifetime (E). This leads to pathways with different q‐values, in addition to weighting of the signal by T2 FIGURE S2 Processing pipeline for (A) 2 samples with different diffusion properties but identical relaxation times and (B) identical diffusion properties but different T1 values. Experimental DW‐SSFP data are acquired at multiple flip angles (i, dots) and converted into ADC estimates (ii, dots) (Equation 2, main text). To eliminate the effects of S0, we fit to the DW‐SSFP signal attenuation. The DW‐SSFP signal model incorporating a gamma distribution of diffusivities (Equation 4, main text) is subsequently fit to the ADC estimates at multiple flip angles (by comparing to Equation 2, main text) to determine Dm and Ds (iii). From Equation 3 in the main text and our fitted values of Dm and Ds, we can simulate the estimated ADC with b‐value for a DW‐SE sequence by making comparisons with the DW‐SE signal under the Stejskal‐Tanner model (S=S0exp-bD). From this, we can define an equivalent DW‐SE b‐value, which gives rise to the same ADC estimate at each DW‐SSFP flip angle (iv). Our ADC estimates with DW‐SSFP can be subsequently plotted ve [file MRM-84-873-s001.pdf]

Supporting Information

Supporting Figures

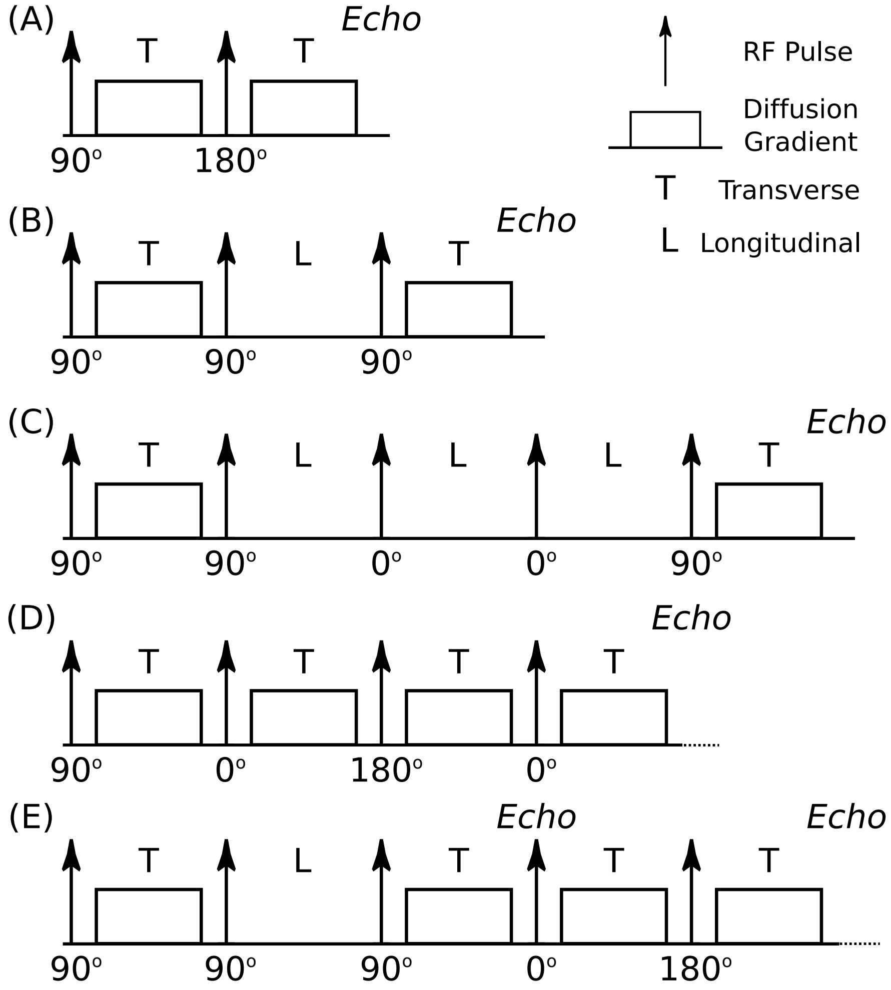

Figure S1: In DW-SSFP, repeated application of RF pulses decomposes the magnetization into a series of coherence pathways, which are sensitized to the diffusion gradient during transverse periods. Here we show 5 example coherence pathways. The spin-echo pathway (A), stimulated-echo pathway (B) and long stimulated-echo pathway (C) only survive for 2 TRs in the transverse plane, the condition for the two-transverse-period approximation (1). These pathways all experience the same q-value, but have different diffusion times, defined as $\Delta = 1 \cdot \text{TR}$ (A), $2 \cdot \text{TR}$ (B), and $4 \cdot \text{TR}$ (C). For the full Buxton model (1), this condition is no longer required, and pathways can experience cumulative sensitization to the diffusion gradients over multiple TRs, such as the spin-echo pathway in (D), in addition to pathways that generate multiple echoes over their lifetime (E). This leads to pathways with different q-values, in addition to weighting of the signal by T_2

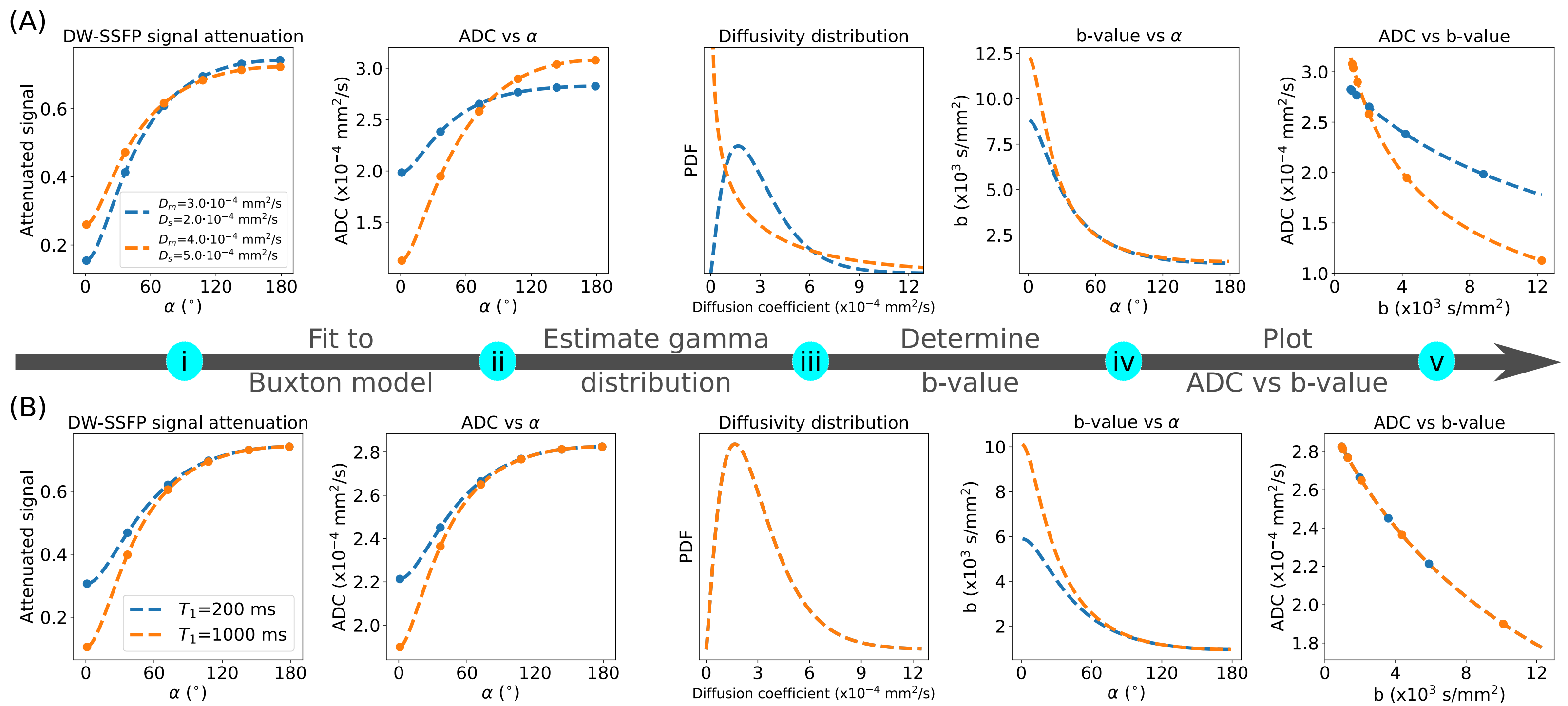

Figure S2: Processing pipeline for (A) 2 samples with different diffusion properties but identical relaxation times and (B) identical diffusion properties but different T_1 values. Experimental DW-SSFP data are acquired at multiple flip angles (i, dots) and converted into ADC estimates (ii, dots) (Equation 2, main text). To eliminate the effects of S_0, we fit to the DW-SSFP signal attenuation. The DW-SSFP signal model incorporating a gamma distribution of diffusivities (Equation 4, main text) is subsequently fit to the ADC estimates at multiple flip angles (by comparing to Equation 2, main text) to determine D_m and D_s (iii). From Equation 3 in the main text and our fitted values of D_m and D_s, we can simulate the estimated ADC with b-value for a DW-SE sequence by making comparisons with the DW-SE signal under the Stejskal-Tanner model ($S = S_0 \exp(-bD)$). From this, we can define an equivalent DW-SE b-value which gives rise to the same ADC estimate at each DW-SSFP flip angle (iv). Our ADC estimates with DW-SSFP can be subsequently plotted versus an effective b-value (v). In (A), this leads to distinct evolution of ADC with effective b-value for the 2 samples (v). However, in (B), the signal evolution is identical (v), despite having a different ADC evolution verses flip angle (ii), reflecting differences in the weighting of the different coherence pathways due to relaxation, leading to different effective b-values along the ADC curve (v, dots)

Supporting Tables

Turbo inversion recovery (TIR) - T_1

Resolution	0.65 x 0.65 x 1.30 mm^3
TR	1000 ms
TE	12 ms
TIs	60, 120, 240, 480, 940 ms
BW	170 Hz/pixel

Turbo spin echo (TSE) - T_2

Resolution	0.65 x 0.65 x 1.30 mm^3
TR	1000 ms
TEs	11, 23, 34, 46, 57, 69 ms
BW	163 Hz/pixel

Actual flip angle imaging (AFI) - B_1

Resolution	1.50 x 1.50 x 1.50 mm^3
TRs	7, 21 ms
TE	2.6 ms
BW	263 Hz/pixel

Table S1: Acquisition protocols for the T_1, T_2 and B_1 maps. Before processing, a Gibbs ringing correction was applied to the TIR and TSE data (2). T_1 and T_2 maps were derived assuming mono-exponential signal evolution. The B_1 map was obtained using the methodology described in (3)

Supporting Derivations

The two-transverse-period approximation with a gamma distribution of diffusivities

From Equation 1 in the main text:

$$S_{\mathrm{SSFP}}(\alpha, T_1, T_2, \mathrm{TR}, q, D) = \frac{-S_0(1-E_1)E_1E_2^2\sin\alpha}{2(1-E_1\cos\alpha)}\left[\frac{1-\cos\alpha}{E_1}A_1 + \sin^2\alpha\sum_{n=1}^{\infty}(E_1\cos\alpha)^{n-1}A_1^{n+1}\right], \quad \text{[S1]}$$

where S_0 is the equilibrium magnetization, $E_1 = e^{-\frac{\mathrm{TR}}{T_1}}$, $E_2 = e^{-\frac{\mathrm{TR}}{T_2}}$, α is the flip angle, n is the number of TRs between the two transverse-periods for a given stimulated-echo, $A_1 = e^{-q^2\cdot\mathrm{TR}\cdot D}$, D is the diffusion coefficient and $q = \gamma G\tau$, where γ is the gyromagnetic ratio, G is the diffusion gradient amplitude and τ is the diffusion gradient duration. Separating this expression into spin-echo (SE) and stimulated-echo (STE) pathways:

$$S_{\mathrm{SE}} = \frac{-S_0(1-E_1)E_2^2\sin\alpha(1-\cos\alpha)}{2(1-E_1\cos\alpha)}\cdot e^{-q^2\cdot\mathrm{TR}\cdot D}, \quad \text{[S2]}$$

and:

$$S_{\mathrm{STE}} = \frac{-S_0(1-E_1)E_1E_2^2\sin\alpha}{2(1-E_1\cos\alpha)}\cdot\sin^2\alpha\cdot\sum_{n=1}^{\infty}\left[(E_1\cos\alpha)^{n-1}\cdot e^{-q^2\cdot(n+1)\cdot\mathrm{TR}\cdot D}\right]. \quad \text{[S3]}$$

SE term

Integrating over the SE term with a gamma distribution of diffusivities:

$$S_{\mathrm{SE},\Gamma} = \frac{-S_0(1-E_1)E_2^2\sin\alpha(1-\cos\alpha)}{2(1-E_1\cos\alpha)}\cdot\int_0^{\infty}e^{-q^2\cdot\mathrm{TR}\cdot D}\rho(D;D_m,D_s)dD, \quad \text{[S4]}$$

where $\rho(D;D_m,Ds)$ is the gamma distribution with mean D_m and standard deviation D_s over D. From Equation 3 in the main text:

$$\int_0^{\infty}e^{-q^2\cdot\mathrm{TR}\cdot D}\rho(D;D_m,D_s)dD = \left[\frac{D_m}{D_m+q^2\cdot\mathrm{TR}\cdot D_s^2}\right]^{\frac{D_m^2}{D_s^2}}. \quad \text{[S5]}$$

Therefore:

$$S_{\mathrm{SE},\Gamma} = \frac{-S_0(1-E_1)E_2^2\sin\alpha(1-\cos\alpha)}{2(1-E_1\cos\alpha)}\cdot\left[\frac{D_m}{D_m+q^2\cdot\mathrm{TR}\cdot D_s^2}\right]^{\frac{D_m^2}{D_s^2}}. \quad \text{[S6]}$$

STE term

Integrating over the STE term with a gamma distribution:

$$S_{\mathrm{STE},\Gamma} = \frac{-S_0(1-E_1)E_1E_2^2\sin\alpha}{2(1-E_1\cos\alpha)} \cdot \sin^2\alpha \cdot \sum_{n=1}^{\infty}(E_1\cos\alpha)^{n-1} \cdot \int_0^{\infty} e^{-q^2\cdot(n+1)\cdot\mathrm{TR}\cdot D}\rho(D;D_m,D_s)dD. \quad \text{[S7]}$$

Evaluating the summation term, considering Equation 3 in the main text:

$$\begin{aligned}&\sum_{n=1}^{\infty}(E_1\cos\alpha)^{n-1} \cdot \int_0^{\infty} e^{-q^2\cdot(n+1)\cdot\mathrm{TR}\cdot D}\rho(D;D_m,D_s)dD \\ =&\sum_{n=1}^{\infty}(E_1\cos\alpha)^{n-1} \cdot \left[\frac{D_m}{D_m+q^2\cdot(n+1)\cdot\mathrm{TR}\cdot D_s^2}\right]^{\frac{D_m^2}{D_s^2}},\end{aligned} \quad \text{[S8]}$$

Pulling D_m from the numerator and $q^2 \cdot \mathrm{TR} \cdot D_s^2$ from the denominator :

$$= \left(\frac{D_m}{q^2\cdot\mathrm{TR}\cdot D_s^2}\right)^{\frac{D_m^2}{D_s^2}} \cdot \sum_{n=1}^{\infty}\frac{(E_1\cos\alpha)^{n-1}}{\left[\frac{D_m}{q^2\cdot\mathrm{TR}\cdot D_s^2}+(n+1)\right]^{\frac{D_m^2}{D_s^2}}}. \quad \text{[S9]}$$

Rearranging and defining $m = n - 1$:

$$= \left(\frac{D_m}{q^2\cdot\mathrm{TR}\cdot D_s^2}\right)^{\frac{D_m^2}{D_s^2}} \cdot \sum_{m=0}^{\infty}\frac{(E_1\cos\alpha)^{m}}{\left[\left(\frac{D_m}{q^2\cdot\mathrm{TR}\cdot D_s^2}+2\right)+m\right]^{\frac{D_m^2}{D_s^2}}}. \quad \text{[S10]}$$

The summation term is in an equivalent format to the the Lerch Transcendent (4), defined as:

$$\Phi(z,s,a) = \sum_{m=0}^{\infty}\frac{z^m}{(a+m)^s}, \quad \text{[S11]}$$

where $z = E_1\cos\alpha$, $s = \frac{D_m^2}{D_s^2}$ and $a = \frac{D_m}{q^2\cdot\mathrm{TR}\cdot D_s^2} + 2$. Therefore:

$$S_{\mathrm{STE},\Gamma} = \frac{-S_0(1-E_1)E_1E_2^2\sin\alpha}{2(1-E_1\cos\alpha)} \cdot \sin^2\alpha \cdot \left(\frac{D_m}{q^2\cdot\mathrm{TR}\cdot D_s^2}\right)^{\frac{D_m^2}{D_s^2}} \cdot \Phi\left(E_1\cos\alpha, \frac{D_m^2}{D_s^2}, \frac{D_m}{q^2\cdot\mathrm{TR}\cdot D_s^2}+2\right). \quad \text{[S12]}$$

Total signal

Summing the SE and STE terms:

$$S_{\text{SSFP},\Gamma}(\alpha, T_1, T_2, \text{TR}, q, D) = \frac{-S_0(1-E_1)E_1E_2^2 \sin\alpha}{2(1-E_1\cos\alpha)} \cdot \left[\frac{1-\cos\alpha}{E_1} \cdot \left(\frac{D_m}{D_m + q^2 \cdot \text{TR} \cdot D_s^2} \right)^{\frac{D_m^2}{D_s^2}} + \sin^2\alpha \cdot \left(\frac{D_m}{q^2 \cdot \text{TR} \cdot D_s^2} \right)^{\frac{D_m^2}{D_s^2}} \cdot \Phi\left(E_1\cos\alpha, \frac{D_m^2}{D_s^2}, \frac{D_m}{q^2 \cdot \text{TR} \cdot D_s^2} + 2 \right) \right]. \quad \text{[S13]}$$

ADC expression under the two-transverse-period approximation

Summing over Equation 1 in the main text, noting $\sum_{m=0}^{\infty} r^m = \frac{1}{1-r}$, or from (1):

$$S_{\text{SSFP}}(\alpha, T_1, T_2, \text{TR}, q, \text{ADC}) = -\frac{S_0(1-E_1)(1+E_1A_{\text{ADC}})A_{\text{ADC}}(1-\cos\alpha)\sin\alpha}{2(1-E_1\cos\alpha)(1-A_{\text{ADC}}E_1\cos\alpha)} \cdot E_2^2. \quad \text{[S14]}$$

Maintaining terms that depend on ADC:

$$\frac{(1+E_1A_{\text{ADC}})A_{\text{ADC}}}{(1-A_{\text{ADC}}E_1\cos\alpha)} = \frac{S_{\text{SSFP}}(\alpha, T_1, T_2, \text{TR}, q, \text{ADC})}{S_{\text{SSFP}}(\alpha, T_1, T_2, \text{TR}, 0, \text{ADC})} \cdot \frac{1+E_1}{1-E_1\cos\alpha} = S'_{\text{SSFP}}. \quad \text{[S15]}$$

$S_{\text{SSFP}}(\alpha, T_1, T_2, \text{TR}, q, \text{ADC})$ and $S_{\text{SSFP}}(\alpha, T_1, T_2, \text{TR}, 0, \text{ADC})$ can be substituted by diffusion-weighted and non diffusion-weighted data respectively. Multiplying Equation S15 by the denominator:

$$(1+E_1A_{\text{ADC}})A_{\text{ADC}} - S'_{\text{SSFP}} \cdot (1-A_{\text{ADC}}E_1\cos\alpha) = 0. \quad \text{[S16]}$$

Expanding the brackets and reordering:

$$E_1A_{\text{ADC}}^2 + (S'_{\text{SSFP}} \cdot E_1\cos\alpha + 1)A_{\text{ADC}} - S'_{\text{SSFP}} = 0. \quad \text{[S17]}$$

This is a quadratic equation, therefore:

$$A_{\text{ADC}} = \frac{-(S'_{\text{SSFP}} \cdot E_1\cos\alpha + 1) \pm [(S'_{\text{SSFP}} \cdot E_1\cos\alpha + 1)^2 + 4E_1 \cdot S'_{\text{SSFP}}]^{\frac{1}{2}}}{2E_1}. \quad \text{[S18]}$$

As E_1 and S'_{SSFP} are positive, the numerator is less than 0 when we consider the negative solution. Considering $A_{\text{ADC}} = e^{-q^2 \cdot \text{TR} \cdot \text{ADC}}$, this would lead to a complex definition of ADC. Therefore:

$$\text{ADC} = -\frac{1}{q^2\text{TR}} \cdot \ln\left[\frac{-(S'_{\text{SSFP}} \cdot E_1\cos\alpha + 1) + [(S'_{\text{SSFP}} \cdot E_1\cos\alpha + 1)^2 + 4E_1 \cdot S'_{\text{SSFP}}]^{\frac{1}{2}}}{2E_1} \right]. \quad \text{[S19]}$$

For a Gamma distribution of diffusivities, by comparing Equation S13 to Equations S14 and S15:

$$S'_{\mathrm{SSFP},\Gamma} = \left(\frac{D_m}{D_m + q^2 \cdot \mathrm{TR} \cdot D_s^2}\right)^{\frac{D_m^2}{D_s^2}} + E_1 \cdot (1 + \cos\alpha) \cdot \left(\frac{D_m}{q^2 \cdot \mathrm{TR} \cdot D_s^2}\right)^{\frac{D_m^2}{D_s^2}} \cdot \Phi\left(E_1 \cos\alpha, \frac{D_m^2}{D_s^2}, \frac{D_m}{q^2 \cdot \mathrm{TR} \cdot D_s^2} + 2\right). \qquad \text{[S20]}$$

References

1. Buxton RB. The diffusion sensitivity of fast steady-state free precession imaging. Magn. Reson. Med. 1993. doi: 10.1002/mrm.1910290212.

2. Kellner E, Dhital B, Kiselev VG, Reisert M. Gibbs-ringing artifact removal based on local subvoxel-shifts. Magn. Reson. Med. 2016. doi: 10.1002/mrm.26054.

3. Yarnykh VL. Actual flip-angle imaging in the pulsed steady state: A method for rapid three-dimensional mapping of the transmitted radiofrequency field. Magn. Reson. Med. 2007. doi: 10.1002/mrm.21120.

4. Erdilyi A, Magnus W, Oberhettinger F, Tricomi FG. Higher transcendental functions, vol. 1. Bateman Manuscr. Proj. McGraw-Hill, New York 1953:27–31.
